# Predictors and factors associated with stunting among under- five-year children: a cross-sectional population-based study in Rwanda of the 2014–2015 demographic and Health Survey

**DOI:** 10.4314/ahs.v22i4.73

**Published:** 2022-12

**Authors:** Joselyne Rugema, Joselyne Mukantwari, Innocent Twagirayezu, Marie Jeanne Tuyisenge, Reverie Rutayisire, Godfrey Katende

**Affiliations:** 1 University of Rwanda College of Medicine and Health Sciences, School of Nursing and Midwifery; 2 Sultan Qaboos University, Nursing; New York University, Rory- meyers, School of Nursing and Midwifery; University of Rwanda College of Medicine and Health Sciences, School of Nursing and Health Sciences

**Keywords:** Predictors, Stunting, children, under five years, Rwanda

## Abstract

**Background:**

Globally, stunting affects 159 million Under-five-years-old (U-5) children. Stunting affects the physical, mental, and cognitive development of children increasing the risk of suffering and death. This paper aimed to determine the predictors and factors associated with stunting among under-five children in Rwanda.

**Methods:**

We retrieved data from the Rwanda Demographic and Health Survey (RDHS) 2014–2015 data set. A sample of 3599 U-5 eligible children with complete and valid anthropometric data was taken. Stata software was used to analyze the data extracted using a developed checklist. Descriptive statistics and Logistic regression analysis were performed to test the association between study variables.

**Results:**

Of 3599 U-5 children, 37.5% of children were stunted. The demographic characteristics: age (p< 0.001), sex (p<0.001), and place of residence (p< 0.001) and Household wealth index (p <0.001) were associated with stunting. Age, sex, and household wealth index were predictors of stunting.

**Conclusion:**

Stunting is still a burden in Rwanda. Age and sex were predictors of stunting among children under 5 years of age. Household wealth index was a predictor and significantly associated with stunting among children U-5 years in Rwanda. Investing in the interventions that target maternal and nutrition health support is imperative.

## Introduction

World Health Organization (WHO) defines child Stunting as a condition when a child is short for her/his age compared with height-for-age Z (HAZ) score below minus two standard deviations^1^. Stunting is a result of children suffering from chronic and acute malnutrition secondary to infections and inappropriate diets. Children found to be stunted will present with failure of physical and cognitive development, poor school outcomes, increased risk of suffering, and as well as death^1^.

Globally, more than 159 million under five years old children are stunted with an estimate of 80% found in only 14 low middle-income countries^2^,^3^,^4^. Over 40 percent of the global burden of stunted children is in Africa^5^. Sub-Saharan Africa is also disproportionally affected by stunting with 34% of under-five years children found in the Eastern Africa countries^6^.

In Rwanda, the recorded prevalence of stunting in under-five- years children were 56.8% in 1992, 48.3% in 2000, 51.1% in 2005, 44.2% in 2010, and 38% in 2015^7^. This, therefore, signifies a problem of chronic malnutrition existing in Rwanda^5^.

While available evidence points out that stunting begins in utero and continues until the first two years of life, there are also interventions to prevent its occurrence and associated complications^8^. It is important to recognize that stunting throughout the first two years of life presents detrimental and irreversible consequences such as impaired neurocognitive development, decreased educational performance, reduced economic productivity in adulthood, and increased morbidity and mortality^4^. Other risk factors associated with stunting are: being male, children ages 6–23 months and 24–59 months, low birth weight, low maternal height, low education level of mothers, a history of not taking deworming medicine during pregnancy, and low economic status of households^6^,^8^. Additionally, the major contributors to childhood stunting include the causal influences of household and family factors such as; inadequate complementary feeding, breastfeeding, and infection, as well as contextual factors of community and societal impacts 8,9. Both the environmental^10^ and the health care system^11^ factors also contribute to stunting. Interventions such as micronutrients supplementation during pregnancy, counselling about Infant and young child nutrition, the antenatal supplementation of balanced energy-protein molecules, mothers from endemic regions receiving presumptive treatment of Malaria during pregnancy, providing vitamins, all in the effort to reduce the burden of stunting in Rwanda are being implemented^12^.

Wealth is described as a household characteristic that has a significant effect on health^12^. The wealth index is a traditional indicator and a measure of household economic status for those countries that lack reliable data on income and expenditures^12^. Rwanda in particular is a small, high populated, and locked country with around 62% of the population living on less than US$1.25 per day, faces a burden of more than a third of its population experiencing food insecurity due to low agricultural productivity^13^. This, therefore, translates into a high cost of food requiring a significant investment in low-income earning families leading to the unaffordability of nutritious foods^12^. Interventions intended to finance underlying determinants of malnutrition in Rwanda in collaboration with international donors and partners are being implemented through various programs to address poverty among the communities^13^. These include but are not limited to Hinga Weze-Cultivate for Better Production, Twiyubake-the Improved Services for Vulnerable Populations, Gimbuka- Caritas Rwanda USAID, Gikuriro-Integrated Nutrition and WASH Activity, Feed the future Rwanda, and Orange-Fleshed Sweet Potato^13^. Other projects are Gira inka munyanrwanda, all these in the effort to improve family income and nutrition. Therefore, reaching the stunting target is feasible by addressing households' wealth index and other associated factors to stunting in the U-5^12^,^13^,^14^. This paper was set to determine the predictors and factors associated with stunting among U-5 children in Rwanda.

## Methods

### Design

This study used a cross-sectional quantitative design to determine the predictors and factors associated with stunting among under- five-year children in Rwanda. Secondary data analysis of the 2014–2015 of the Rwanda Demographic and Health Survey (RDHS) 15 report in 30 districts of Rwanda was conducted.

### Study population

The study's target population was all children under five years of age who completed the criteria under consideration by the Rwanda Demographic and Health Survey of 2014–15. We used a Children's (KR) dataset for analysis. The dataset included children and their mothers who participate in the study.

For eligibility, only those children, whose weight and height were taken and recorded at the time of the survey were included in the study. A weight of less than 2.5kg and those self-reported by the mothers to be small in size were also included in the study. When a child's birth weight was taken and is below 2.5kg without height recorded was excluded

### Sample size

The survey included all children in Rwanda with a total population of 7856 children. The research team purposively selected the under-five children's nutritional variables in the Children's Recode (KR) dataset. Using the eligibility criteria, a total of 3599 children with complete and valid anthropometric data constituted the sample.

### Data collection

Data were retrieved from the Rwanda Demographic Health Survey (2014–2015). The RDHS followed a two-stage sample design with the use of cross-sectional surveys during data collection. Before data collection, the research team developed and agreed on using a checklist to use as a data collection tool. The data collection tool (Checklist) had all the variables needed for secondary analysis. The variables included in the data collection tool were: demographic characteristics, wealth index, stunting status among others.

### Measurements

The checklist developed included: demographic characteristics of mainly household children under five. Demographic characteristics were: age in months, sex of the child, and place of residence (urban vs rural). The data was taken from the Children's Recode (KR) dataset.

Stunting status: In this study, stunting status was measured by weight to height measurements recorded in the data set. A weight below 2.5kg and the corresponding height recorded was taken for further processing to determine stunted and non- stunted children.

Wealth index: Using the household questionnaire in the RDHS^15^, the data on the wealth index was extracted for this measure. The data extracted were related to access to electricity, source of drinking water, types of toilet facilities, type of roofing and flooring materials, and ownership of various modern durable goods. The wealth index was categorized into 5 quantile levels ranging from lowest to highest. The study's categories range from poorest to Richest quantile levels (A combined wealth index has a mean of zero and standard deviation of 1)^15^.

### Data analysis

The data analysis was done using Stata software (StataCorp. 2013. Stata Statistical Software: Release 13. College Station, TX: StataCorpLP). The demographic characteristics, wealth index, and stunting status data were analysed descriptively and presented in frequencies and percentages. Multivariate regression analysis was also undertaken on all the variables to determine the associated factors to stunting among U-5 children using a chi-square test. Odds ratio (OR) with 95% confidence interval (CI) was used to evaluate the factors associated with stunting status and other variables. Both unadjusted and adjusted ORs were calculated and analysed.

### Ethical considerations

The data used in this study are based on secondary data obtained with permission from MEASURE DHS Organization and was downloaded from the Demographic and Health Surveys (DHS) online archive. The “ICF International Institutional Review Board” approves all surveys used by the DHS to guarantee that the surveys abide by the “U.S Department of Health and Human Services” regulations to protect human subjects. DHS uses firm measurements to preserve the privacy and confidentiality of the participants and their household members. The participants were asked to accept or deny a consent form. The form informed the participants about the purpose of the survey, expected duration, procedures, potential risks and benefits, and contact information if the participants had more questions or wanted more information. The participants are also informed that participation is voluntary and that they can withdraw at any point. If the participant is a minor, the guardian or parent must approve the consent form before participating in the survey. The original DHS data were collected with approval from the Inner-city (ICF) International's Institutional Review Board and national ethical guidelines. Mothers were given objectives, procedures, potential risks, and benefits before enrolling their children in the study. Informed consent was read in the local language, and a copy was given to the household upon request. We requested the approval and accessibility for using the Rwanda Demographic and Health Survey 2014–15 dataset for this study via the DHS website and received it 24 hours after the request.

## Results

### Demographic characteristics

The findings showed that more than half (57.7%) of the participants were aged between 24 and 59 months of age. Slightly more than half (50.4%) were males with the majority (83.2%) of the participants residing in rural settings (Table 1).

**Table 1:** Socio-demographic characteristics of children 0–59 months

Characteristics	n	%
Stunting		
Not stunted	2260	62.5
Stunted	1339	37.5
Age category(months)		
<6 months	353	9.7
11–23	1171	32.6
24–59	2091	57.7
Sex of child		
Male	3978	50.4
Female	3878	49.6
Place of residence		
Urban	1725	16.8
Rural	6131	83.2
Wealth index		
Poorest	1893	24.2
Poorer	1643	21.7
Middle	1479	19.7
Richer	1340	17.3
Richest	1501	17.1

### Stunting status

The study revealed that 37.5 % of the participants were stunted at the time of the survey (Table 1).

### Wealth index

The results showed that the majority of the participants were drawn from the poorest (24.2%) and poorer (21.7%) quantile levels (Table 1).

### Association between social-demographic characteristics, household wealth index and stunting status

The results showed that there was a significant association between U-5 children stunting and demographic characteristics, namely age (p< 0.001), sex (p<0.001), and place of residence (p< 0.001), similarly, household wealth index (p <0.001) was associated with stunting (Table 2).

**Table 2:** Association between demographic characteristics, household wealth index of under 5 children and stunting status

	Stunted		
	
	n	%	p-value
Age category(months)			<0.001*
<6 months	36	1	
11–23	421	11.7	
24–59	882	24.8	
Sex of child			<0.001*
Male	767	21.4	
Female	572	16.1	
Place of residence			<0.001*
Urban	188	3.9	
Rural	1151	33.6	
Wealth index			<0.001*
Poorest	421	11.8	
Poorer	346	10.1	
Middle	255	7.3	
Richer	180	5	
Richest	137	3.4	

CI= 95%

* Significant variables with a p-value ≥ 0.05

### Predictors and correlates of stunting

The multivariate analysis with an adjusted model revealed that demographic characteristics were predictors of stunting. Under-five children aged between 11 and 23 months were five times (OR 5.1, p< 0.001) more likely to be stunted. Also, children aged between 24 and 59 months were seven times (OR 6.7, p<0.001) more likely to be stunted. Female children (OR=0.65, p< 0.001) were less likely to be stunted. Contrarily, the place of residence was not a predictor of stunting using the adjusted model. About the household wealth index, children coming from the middle (OR 0.6, p<0.00), richer (OR 0.4, p<0.001), and richest (OR 0.3, p<0.001) incomes were less likely to be stunted compared to their counterparts in the poorer and poorest income (p> 0.05) (Table 3).

**Table 3:** Multivariate analysis of Demographic characteristics, household wealth index and stunting status

	Unadjusted model		Adjusted model	

Factors	OR (95%CI)	p-value	OR (95%CI)	p-value
Age category				
<6 months	1		1	
11–23	4.9(3.3–7.2)	<0.001	5.1(3.4–7.6)	<0.001*
24–59	6.9(4.4–9.6)	<0.001	6.7(4.5–10.1)	<0.001*
Sex of child				
Male	1		1	
Female	0.7(0.6–0.8)	<0.001	0.6(0.6–0.7)	<0.001*
Place of residence				
Urban	1			
Rural	2.2(1.7–2.8)	<0.001		
Wealth index				
Poorest	1		1	
Poorer	0.9(0.7–1.1)	0.361	0.7(0.7–1.1)	0.201
Middle	0.6(0.5–0.8)	<0.001	0.6(0.5–0.8)	<0.001*
Richer	0.5(0.4–0.6)	<0.001	0.4(0.3–0.6)	<0.001*
Richest	0.3(0.2–0.4)	<0.001	0.3(0.2–0.4)	<0.001*

CI =95%

* Significant variables with a p-value ≤ 0.05

## Discussion

The study showed the prevalence of stunting is still a big problem comparable to the national stunting rate. The findings are consistent with other similar findings conducted in Ethiopia and Nigeria and those of a systematic review done in 137 developing countries 10,17,18.

In our study, a large proportion of children were found to be in rural settings with an increased risk of stunting among children of advanced age. Children between 24–59 months were seven times more likely to be stunted than those in earlier stages. Similarly, our study also found that female children were less likely to be stunted than males. The findings are consistent with findings in other studies that confirm that being male and children with ages of 24–59 months posed greater risks to stunting 8. In fact, after weaning a child from breastfeeding, commonly at 2^nd^ birthday in Rwandan culture, the child may be regarded as old enough to be left alone with his or her siblings or other caretakers while the mother is back to work (informal and formal) depending on the occupation of the mother. Also, from the available evidence, we recognize that as children grow up, there is an increase in body demand for nourishment. This also means increased intake of nourishing foods both in quantity and quality which may not be possible depending on the economic status of the household. There is also the likelihood that mothers of such children may not have adequate complementary feeds after breastfeeding, which increases the risk of children acquiring early childhood infections^8^,^9^,^12^. The findings are in line with the available evidence that supports the fact that a greater risk of stunting is associated with the low economic status of households 8,12.

In this study, it was revealed that the household wealth index was a predictor of stunting in which children of families from poorest to poorer families were more likely to be stunted than those from middle to richest families. This is similar to findings of studies conducted in Bangladesh^19^, Ethiopia^18^, Myanmar^9^, and Indonesia^20^, which demonstrated that pro-poor socioeconomic inequality contributed to stunting in the U-5 children. Children from low-income families may have disadvantages of the likelihood to experience food shortages as an example leading to food insecurity, inappropriate feeding that in the end causes malnutrition and consequently leading to stunting^7^,^8^,^12^ among other forms of malnutrition. The findings are also supported by the shreds of evidences that demonstrate that stunted children may present with irreversible consequences ranging from neurocognitive development to decreased productivity in adulthood among others^4^, ^8^,^9^.

Additionally, families in low household wealth index categories may also have disadvantages related to poor maternal and nutrition health which are adequately explicated as greater risks associated with stunting in numerous studies^6^,^8^,^21^,^22^.

In moving forward to address the existing stunting problem in Rwanda, our study recognizes the government and its partners' efforts in the various interventions implemented^12^, however, there should be exceptional follow-up on food supplementation to children in the weaning period from poor and poorest families. This can be achieved by coordinating investments that support nutrition interventions as well as supportive policy environment^12^. Also, in-country projects that focus on improving households' incomes need to be enhanced and scaled up. It is envisaged that saving children's lives and building human capital drives economic growth thereby improving nutrition and tackling the stunting burden^12^.

## Limitation of the study

This study presents the following limitations which are related to the methodological approach. The study employed a secondary analysis of data. This means that this study could inherit some limitations during data collection and analysis including missing data. There are also incidences where data was collected from mothers as self-reported, this could introduce self-reported bias during data collection especially on household wealth index data collection tool leading to misclassification of the household wealth index. Therefore, all these limitations may present a problem of generalization of results of this study to other settings.

## Conclusion

Stunting is still a big burden in Rwanda. Demographic characteristics such as age and sex were predictors of stunting among children under 5 years of age. Household wealth index was associated with stunting among children U-5 years in Rwanda. Investing in interventions that target maternal and nutrition health support will reduce the burden of stunting not only in Rwanda but also in other low and middle-income countries. Improving household incomes would have a long-lasting impact on especially families with low household wealth index. Further studies are needed to determine the impact of these interventions on other malnutrition disorders.
